# LncRNA Nuclear-Enriched Abundant Transcript 1 Regulates Atrial Fibrosis via the miR-320/NPAS2 Axis in Atrial Fibrillation

**DOI:** 10.3389/fphar.2021.647124

**Published:** 2021-04-15

**Authors:** Huangdong Dai, Naishi Zhao, Hua Liu, Yue Zheng, Liang Zhao

**Affiliations:** Department of Cardiovascular Surgery, Shanghai Chest Hospital, Shanghai Jiao Tong University, Shanghai, China

**Keywords:** atrial fibrillation, atrial fibrosis, NEAT1, miR-320, NPAS2

## Abstract

Atrial fibrosis is a key contributor to atrial fibrillation (AF). Long non-coding ribonucleic acids (lncRNAs) were demonstrated to exhibit a key role in fibrotic remodeling; however, the function of nuclear-enriched abundant transcript 1 (NEAT1) in atrial fibrosis remains unclear. In the present study, we showed that NEAT1 was upregulated in atrial tissues of AF patients and was positively related to collagen I (coll I) and collagen III (coll III) expressions. Furthermore, the deletion of NEAT1 attenuated angiotensin II (Ang II)-caused atrial fibroblast proliferation, migration, and collagen production. We further observed that NEAT1 knockdown improved Ang II caused mouse atrial fibrosis in *in vivo* experiments. Moreover, we demonstrated that NEAT1 could negatively regulate miR-320 expression by acting as a competitive endogenous RNA (ceRNA). miR-320 directly targeted neuronal per arnt sim domain protein 2 (NPAS2) and suppressed its expression. We observed that NEAT1 exerted its function via the miR-320–NPAS2 axis in cardiac fibroblasts. These findings indicate that NEAT1 exerts a significant effect on atrial fibrosis and that this lncRNA is a new potential molecular target for AF treatment.

## Introduction

Atrial fibrillation (AF) is the most common arrhythmia encountered in clinical practice and a main cause of stroke (Chiang et al., 2014). Accumulating evidence has demonstrated that atrial fibrosis serves as a key contributor to AF (Xu et al., 2018). Atrial fibrosis is a marker of structural reconstruction and is seen as a substrate for AF progression (Dzeshka et al., 2015). Advanced atrial fibrosis is related to frequent episodes of AF, conversion of arrhythmias to permanent types, and antiarrhythmic drug therapy is less effective (Corradi, 2014; Dzeshka et al., 2015). AF is a major public health tissue; but no effective means for AF prevention exist so far. Therefore, it is important to further elucidate the pathogenesis of AF.

Long non-coding RNAs (lncRNAs) are a class of RNAs (>200 nucleotides) that are vital for regulating gene function and various cellular processes (Qin et al., 2019). Abundant evidence has confirmed that lncRNAs participate in the progression of cancer, chronic obstructive pulmonary disease, cardiovascular disease, and systemic lupus erythematosus (Jiang et al., 2020; Shen et al., 2020; Wu et al., 2020; Zhang et al., 2020). However, so far, only few lncRNAs have been identified to be associated with cardiac fibrosis. For example, PVT1 facilitates atrial fibrosis through regulating miR-128-3p–SP1 in patients with AF (Cao et al., 2019); knockdown of KCNQ1OT1 attenuates Ang II caused AF (Shen et al., 2018); NRON relieves atrial fibrosis via enhancing the NFATc3 phosphorylation (Wang et al., 2019). Reportedly, lncRNA NEAT1 affects fibrosis of organs, such as liver fibrosis (Jin et al., 2019) and renal fibrosis (Huang et al., 2019); however, the functional role and specific mechanism of NEAT1 in atrial fibrosis still completely unclear.

Circadian rhythms occur around a 24 h oscillation in behavior and physiology associated with the solar day, which exist in essentially all tissue and cell types of the organism (Eckel-Mahan and Sassone-Corsi, 2013; Hurley et al., 2016). Previous research reports that dysregulation of some circadian genes, such as Bmal1 and Clock, contributes to atrial fibrogenesis (Goetze et al., 2010; Choudhury et al., 2015). Neuronal PAS domain protein 2 (NPAS2), one of the core circadian molecules that has been shown to promote hepatocarcinoma cell proliferation, contributed to liver fibrogenesis (Yang et al., 2019). But its role in atrial fibrosis remains unclear. In the present study, we observed that NEAT1 was increased in right atrial tissues of AF patients and was positively related to coll I and coll III expressions. We revealed that NEAT1 knockdown reduced Ang II caused atrial fibroblast proliferation and migration. Moreover, we observed that NEAT1 exerted its function via the miR-320/NPAS2 axis in cardiac fibroblasts. The above findings suggest that NEAT1 exerts a significant effect on atrial fibrosis and that this lncRNA is a new potential molecular target for AF treatment.

## Materials and Methods

### Patients and Tissue Samples

Patients undergoing cardiac valve replacement (The cases of mitral valve replacement were excluded) at Shanghai Chest Hospital (Shanghai, China) were joined in our research and were split into the AF group (*n* = 15) and sinus rhythm (SR) group (*n* = 13), according to preoperative electrocardiogram examination and medical history. Patients with persistent atrial fibrillation were included in our study. Patients with previous coronary atherosclerotic heart disease, chronic pulmonary heart disease, infective endocarditis, hyperthyroidism, severe dysfunction of liver and kidney, and malignant tumors were excluded. All patients in the preoperative period of 6 months without applying any type of angiotensin II receptor blockers and angiotensin converting enzyme inhibitors. Superior vena cava intubation was placed in the right auricle, and the right auricle extracted during the procedure was collected for this study. The right atrium was carefully cleaned with normal saline to remove blood, and the adipose tissue was carefully pruned and removed for use in this study. This research protocol was permitted by the ethics committee of Shanghai Chest Hospital, and written informed consent was obtained from each patient.

### Bioinformatics Analysis

Potential NEAT1 and miR-320 binding sites were predicted using starBase v2.0 (http://starbase.sysu.edu.cn/starbase2/mirLncRNA.php), and miR-320 and NPAS2 binding sites were predicted using Targetscan (http://www.targetscan.org/vert_72/).

### qRT–PCR Analysis

Total RNA was obtained from indicated cells or tissues with Trizol (Invitrogen, Carlsbad, CA, United States). The ratio of the optical density of RNA at 280 and 260 nm was measured by ultraviolet spectrophotometer, and the determination value was between 1.8 and2.0. RNAs were converted into cDNA using the RevertAid First Strand cDNA Synthesis Kit (Thermo Fisher Scientific, Waltham, MA, United States). qRT–PCR was carried out tharough a LightCycler 480 real-time PCR system using SYBR® Premix Ex Taq™ (Takara Bio, Inc., Dalian, China). GAPDH or U6 was functioned as the internal control. The 2^−ΔΔCt^ method was performed to analyze the relative expression level of each gene. The sequence of primers is shown in Table 1.

**TABLE 1:** Primers used for qRT–PCR.

Gene or Primer name	Primer sequence (5′-3′)
*NEAT1-human*	F: CCT​GCC​TTC​TTG​TGC​GTT​TC
	R:CTTGTACCCTCCCAGCGTTT
*NEAT1-mouse*	F: GGG​AAG​GGT​GAC​ATT​GAA​AA
	R: CTC​CCC​AGC​TTC​ACT​TCT​TG
*Coll I-human*	F: GAG​AGC​ATG​ACC​GAT​GGA​TT
	R: CCT​TCT​TGA​GGT​TGC​CAG​TC
*Coll I-mouse*	F: GCT​CCT​CTT​AGG​GGC​CAC​T
	R: CCA​CGT​CTC​ACC​ATT​GGG​G
*Coll III-human*	F: GGT​CCT​CCT​GGA​ACT​GCC​GGA
	R: GAG​GAC​CTT​GAG​CAC​CAG​CGT​GT
*Coll III-mouse*	F: TGA​ATG​GTG​GTT​TTC​AGT​TCA​G
	R: GGT​CAC​TTG​CAC​TGG​TTG​ATA​A
*miR-320-mouse*	F: AAA​AGC​TGG​GTT​GAG​AGG​A
	R: TCC​TCT​CAA​CCC​AGC​TTT​T
*NPAS2-mouse*	F: CGC​AGA​TGT​TCG​AGT​GGA​AAG
	R: GTG​CAT​TAA​AGG​GCT​GTG​GAG
*GAPDH-human*	F: AGC​AAG​AGC​ACA​AGA​GGA​AG
	R: GGT​TGA​GCA​CAG​GGT​ACT​TT
*GAPDH-mouse*	F: AGA​ACA​TCA​TCC​CTG​CAT​CC
	R: GGT​CCT​CAG​TGT​AGC​CCA​AG
*U6-human*	F: GCGCGTCGTGAAGCGTTC
	R: GTGCAGGGTCCGAGGT
*U6-mouse*	F: TGG​AAC​GCT​TCA​CGA​ATT​TGC​G
	R: AGA​CTG​CCG​CCT​GGT​AGT​TGT

### Western Blotting

Atrial muscle tissues and cardiac fibroblasts were homogenized or lyzed in radioimmunoprecipitation assay buffer containing a cocktail of protease inhibitors (Santa Cruz, CA, United States) in accordance with the manufacturer’s instruction. Protein was quantified using the Bicinchoninic Acid (BCA) Protein Assay Kit and boiled for 10 min at 95°C. Total protein was obtained and loaded into 10% SDS-PAGE and transferred onto PVDF membranes. The primary antibodies used in our study are as follows: anti-coll I (Proteintech Group, Wuhan, China), anti-coll III (Proteintech Group, Wuhan, China), anti-NPAS2 (Thermo Fisher Scientific), and anti-GAPDH (Proteintech). Quantification was performed by measuring the signal intensity using ImageJ (National Institute of Health, Rockville, MD, United States).

### Cell Culture and Cell Treatments

Mouse cardiac fibroblasts were obtained from Ginio Biotechnology (Guangzhou, China), cultured in DMEM containing 10% FBS, and were transfected with the plasmids and stimulated with Ang II (1 μM).

### Plasmid Construction and Cell Transfection

The miR-320 mimic, miR-320 inhibitor, and their negative controls (NC mimic and NC inhibitor) were obtained from GenePharma (Shanghai, China). The sequences as follows: miR-320 mimic (sense: 5′-AAA​AGC​UGG​GUU​GAG​AGG​A-3′) or NC mimic (sense: 5′-UUC​UCC​GAA​CGU​GUC​ACG​UTT-3′); miR-320 inhibitor (sense: 5′- UCC​UCU​CAA​CCC​AGC​UUU​U-3′) or NC inhibitor (sense: 5′-CAG​UAC​UUU​UGU​GUA​GUA​CAA-3′). The coding region of the NPAS2 mRNA was cloned into the pcDNA3.1 (+) vector. The lentiviral vector expressing shRNA targeting NEAT1 was obtained from HANBIO (Shanghai, China). Short-hairpin RNA directed against NEAT1 was constructed in pLKO.1-puro vector to generate NEAT1 shRNA expression constructs, the non-targeting sequence (negative control, shNC) were also synthesized. The sequences of shNEAT1 and shNC were as follows: shNEAT1-F: ccg​gCA​GGA​CTA​GGT​GCG​TAG​TGc​tcg​agC​ACT​ACG​CAC​CTA​GTC​CTG​ttt​ttg and shNEAT1-R: aat​tca​aaa​aCA​GGA​CTA​GGT​GCG​TAG​TGc tcg​agC​ACT​ACG​CAC​CTA​GTC​CTG. Cell transfection was carried out with validated vector and lentivirus packaging vectors (pMD2G and pSAX2) using Lipofectamine 2000 (Invitrogen).

### The CCK-8 Assay

For the CCK-8 assay, transfected cells were plated into 96-well plates, and the medium of each well was replaced with culture media containing 10% CCK-8 at 72 h. The absorbance was measured using a microplate reader at an optical density of 450 nm.

### Cell Migration Assay

The transfected cells were stimulated with or without Ang II. After that, indicated cells were cultured in the upper chamber using serum-free DMEM. Twenty-four hours later, non-migratory cells on top of the membrane were taken out, and membranes containing cells on the bottom were fixed and stained. The migratory cells were counted under a microscope.

### Luciferase Activity Assay

Human embryonic kidney 293 (HEK293) T-cells were co-transfected with reporter plasmids including either the wild-type NPAS2 3ʹUTR (NPAS2-WT) or wild-type NEAT1 containing miR-320 binding site (NEAT1-WT) or mutated NPAS2 3ʹUTR (NPAS2-MT) or mutated NEAT1 (NEAT1-MT) and with either the miR-320 mimic or NC mimic. After 48 h inductions, the luciferase assay was conducted, and the relative luciferase activity was determined.

### The Ang II-Induced Atrial Fibrosis Mouse Model

C57BL/6J mice (6–7 weeks old) were subdivided into the control, Ang II (Ang II, 1200 ng/kg/min, was continuously perfused to mice through a micropump), Ang II/shNEAT1 (shNEAT1 was injected into the mice via the tail vein after 28 days of Ang II stimulation), and Ang II/shNC (shNC was injected into the mice via the tail vein after 28 days of Ang II treatment) groups. After 14 days, right atrial muscle tissues were obtained for research.

### Hematoxylin–Eosin Staining and Masson’s Trichrome Staining

Atrial muscle tissues obtained from different groups were fixed in 4% paraformaldehyde, embedded in paraffin and then sectioned into slices (4 μm thick). The slices were conducted to HE and Masson’s trichrome stainings. The photographs of the stained tissues were captured, and analyze the pathological changes using Image-Pro Plus (version 6.0; Media Cybernetics, Inc., Rockville, MD, United States).

### Statistical Analysis

All data analyses were carried out using Prism 5.0 (GraphPad Software, San Diego, CA, United States), and all data are presented as mean ± SD. The significance of the differences was determined using Student’s *t*-test. Correlation between factors was analyzed using spearman’s correlation coefficient rank test. *p* values < 0.05 were recognized to be significant. Each *in vitro* experiment was performed a minimum of three times, and samples were measured in biological triplicates for each experiment.

## Results

### NEAT1 is Upregulated in Patients With Atrial Fibrillation and is Positively Correlated With coll I and coll III

**FIGURE 1:**
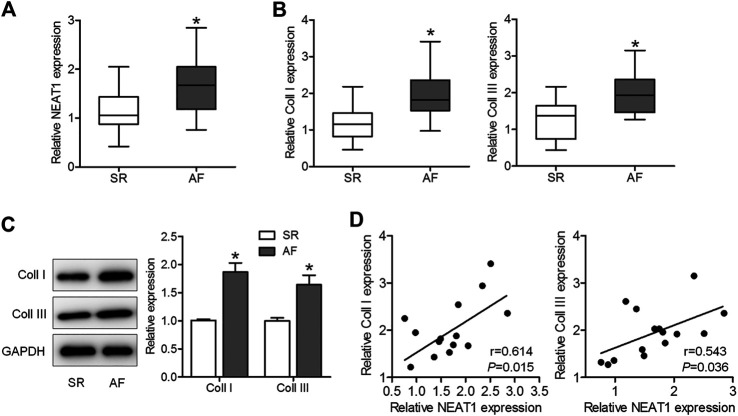
NEAT1 expression is increased in patients with AF and is positively associated with collagen I (coll I) and collagen III (coll III). **(A)** NEAT1 expression in right atrial tissues of patients with AF (n = 15) and those with SR (n = 13) was detected using qRT–PCR analysis. **(B)** The mRNA expression of coll I and coll III in human right atrial tissues was determined using qRT–PCR. **(C)** The protein expression of coll I and coll III in human atrial tissues was determined using western blotting. **(D)** Correlations of NEAT1 with coll I and coll III were analyzed. *p < 0.05. Data represent mean ± SD from three independent experiments.

To explore whether NEAT1 was involved in AF progression, we first determined NEAT1 expression in right atrial tissues of AF patients and SR patients. The results of qRT–PCR observed that NEAT1 expression in right atrial tissues was higher in patients with AF than in those with SR (Figure 1A). Additionaly, we confirmed that coll I and coll III levels were upregulated in the AF group compared to those in the SR group (Figures 1B,C). Additionally, coll I and coll III levels were positively related to NEAT1 expression in right atrial tissues of AF patients (Figure 1D). These observations suggest that NEAT1 participates in atrial fibrosis regulation.

### NEAT1 Deletion Reduces Ang II Caused Murine Cardiac Fibroblast Proliferation, Migration, and Collagen Production

**FIGURE 2:**
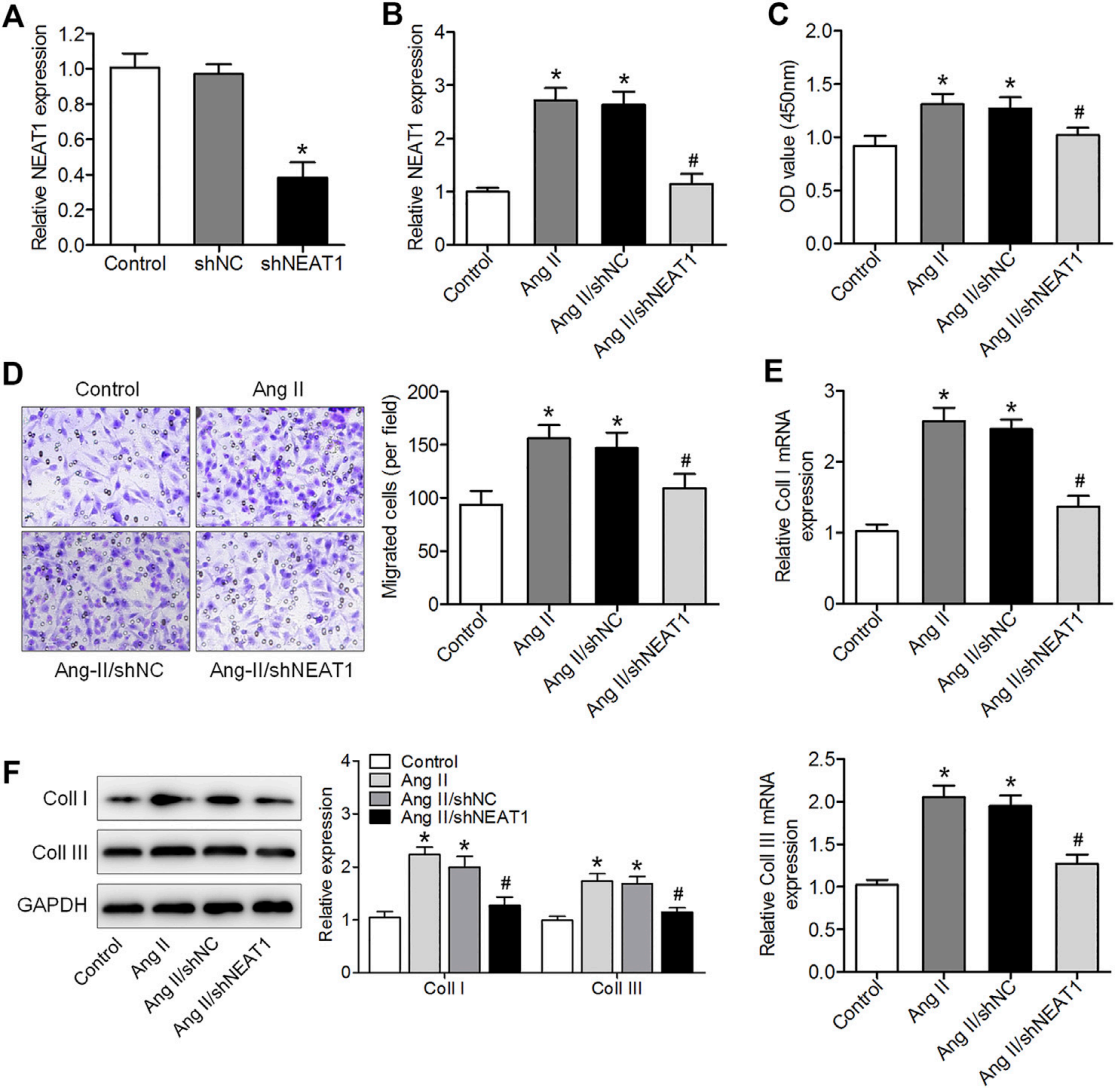
NEAT1 downregulation suppressed Ang II-induced cardiac fibroblast proliferation, migration, and collagen production. **(A)** NEAT1 expression in shNEAT1-or shNC-transfected cardiac fibroblasts was detected using qRT–PCR. **(B)** NEAT1 expression was detected in Ang II-treated cardiac fibroblasts using qRT–PCR. **(C)** Cell proliferation and **(D)** migration were determined using CCK-8 and Transwell assays **(E,F)** mRNA and protein expressions of coll I and coll III were measured using qRT–PCR and western blotting. *p < 0.05 vs. control group; ^#^ *p* < 0.05 vs. Ang II/shNC group. Data represent mean ± SD from three independent experiments.

To explore the effect of NEAT1 on atrial fibrosis, a specific shRNA against NEAT1 gene transcript was designed to knock down NEAT1 in cardiac fibroblasts, and qRT–PCR analysis showed that the expression of NEAT1 in cardiac fibroblasts was reduced by this shRNA (Figure 2A). We found that Ang II increased NEAT1 expression, but this effect was attenuated by NEAT1 shRNA (Figure 2B). The CCK-8 assay showed that NEAT1 downregulation significantly repressed Ang II-induced cell proliferation compared with the shNC-transfected group (Figure 2C). The Transwell migration assay results showed that Ang II treatment promoted the migration ability of cardiac fibroblasts, and this promotion effect was suppressed by shNEAT1 (Figure 2D). Moreover, NEAT1 knockdown attenuated the promoted coll I and coll III expressions after Ang II treatment (Figures 2E,F). These findings revealed that NEAT1 knockdown could inhibit Ang II caused cardiac fibroblast proliferation, migration, and collagen production.

### NEAT1 Positively Regulates NPAS2 Expression Through Sponging miR-320

**FIGURE 3:**
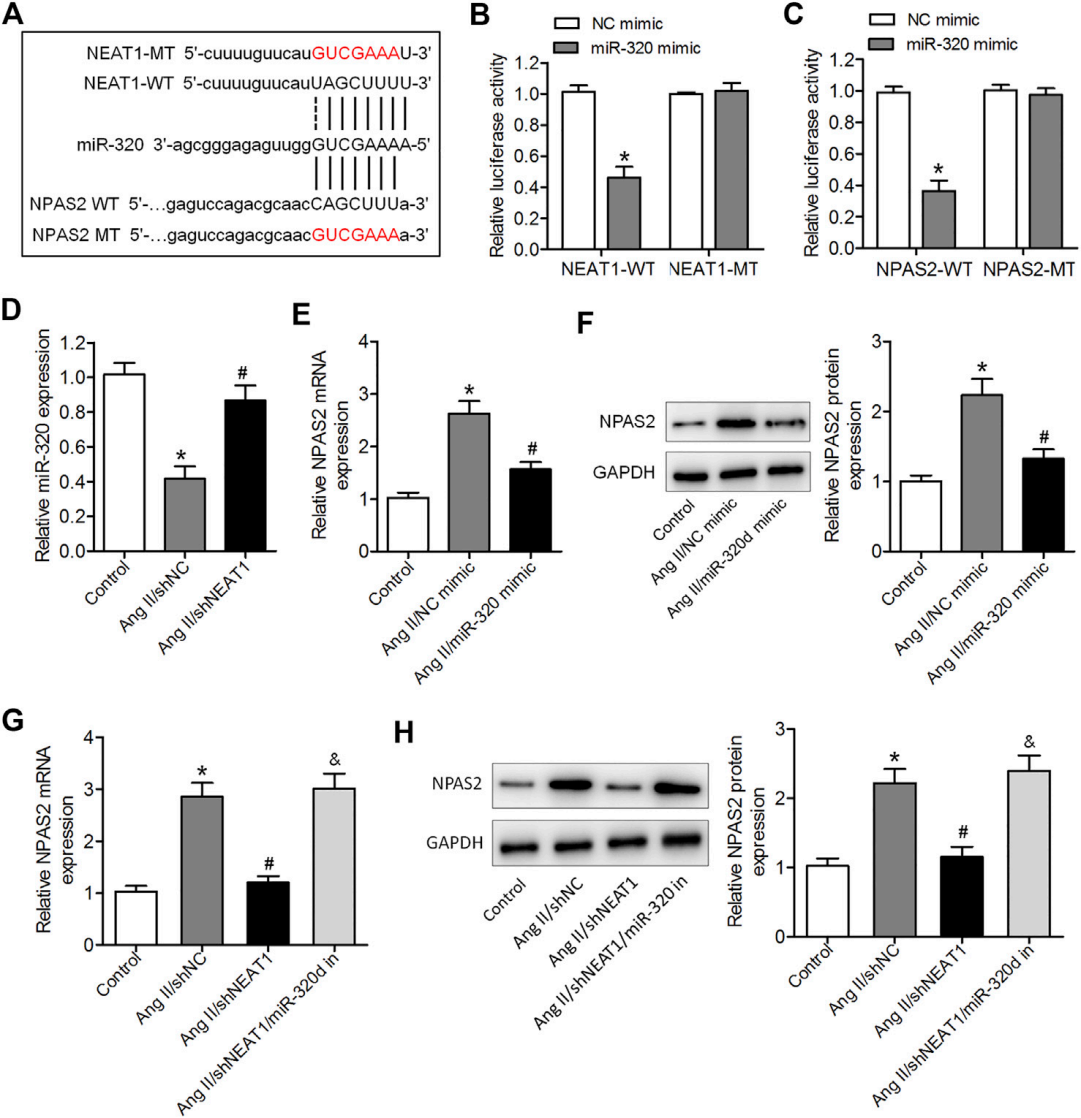
NEAT1 regulated NPAS2 expression through miR-320. **(A)** The predicted miR-320 and NPAS2 binding sites in NEAT1 (NEAT1-WT) and the designed mutant sequence (NEAT1-MT and NPAS2-MT) were indicated. **(B)** The luciferase reporter assay in HEK293T cells co-transfected with NEAT1-WT or NEAT1-MT and NC mimic or miR-320 mimic. **(C)** The luciferase reporter assay in HEK293T cells co-transfected with NPAS2-WT or NPAS2-MT and NC mimic or miR-320 mimic. **(D)** miR-320 expression in Ang II-induced cardiac fibroblasts transfected with shNC or shNEAT1. **(E,F)** mRNA and protein expressions of NPAS2 in Ang II-induced cardiac fibroblasts transfected with NC mimic or miR-320 mimic. **(G,H)** mRNA and protein expressions of NPAS2 in Ang II-induced cardiac fibroblasts transfected with shNEAT1 or co-transfected with shNEAT1 and miR-320 inhibitor were determined. *p < 0.05 vs. NC mimic group or control group; ^#^ *p* < 0.05 vs. Ang II/shNC group; ^&^ *p* < 0.05 vs. Ang II/shNEAT1/miR-320 inhibitor group. Data represent mean ± SD from three independent experiments.

To determine the mechanisms through which NEAT1 exerts its effects on atrial fibrosis, we predicted miR-320 using relevant binding sites of NEAT1, and miR-320 interacted with NPAS2 mRNA 3ʹUTR using bioinformatics databases (Figure 3A). Furthermore, bioinformatics analysis predicted that NEAT1 and NPAS2 mRNAs have the same binding site for miR-320 (Figure 3A). The data of luciferase reporter assay revealed that luciferase activity was inhibited in NEAT1-WT- and miR-320 mimic-co-transfected cells but was unaffected in NEAT1-MT-transfected cells, indicating that miR-320 is a NEAT1-targeting miRNA (Figure 3B). Co-transfection with NPAS2-WT and miR-320 mimic obviously suppressed luciferase activity, whereas the luciferase activity has no changed in co-transfection with NPAS2-MT and miR-320 mimic (Figure 3C). Furthermore, we observed that miR-320 was downregulated in Ang II-treated cardiac fibroblasts, but both parameters were enhanced by NEAT1 deletion (Figure 3D). Ang II-treated cardiac fibroblasts increased NPAS2 expression, but this increase was reduced by miR-320 (Figures 3E,F). Furthermore, NEAT1 knockdown significantly suppressed NPAS2 expression in Ang II stimulated cardiac fibroblasts, and this effect was reversed by miR-320 inhibition (Figures 3G,H). These findings revealed that NEAT1 positively regulates NPAS2 expression by sponging miR-320 in cardiac fibroblasts. NPAS2 overexpression reversed the effects of NEAT1 knockdown on Ang II-induced cardiac fibroblast proliferation, migration, and collagen production.

**FIGURE 4:**
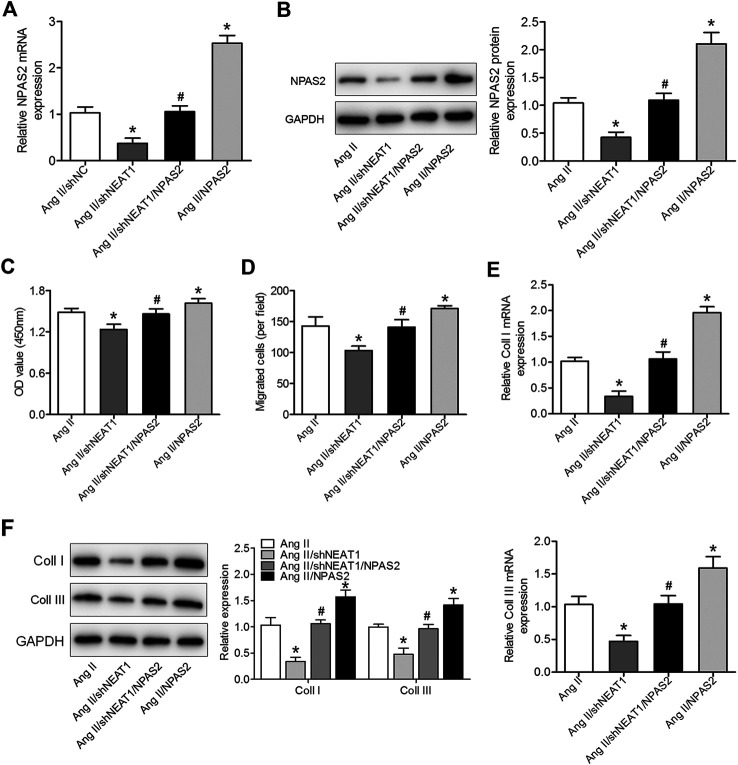
NPAS2 overexpression reversed the effect of NEAT1 knockdown on cardiac fibroblast proliferation, migration, and collagen production under Ang II condition. **(A,B)** mRNA and protein expressions of NPAS2 in cardiac fibroblasts transfected with shNEAT1 or NPAS2 or co-transfected with shNEAT1and NPAS2 under Ang II induction were detected. **(C)** Cell proliferation and **(D)** migration were determined in different groups. **(E,F)** mRNA and protein expressions of coll I and coll III in different groups were determined. *p < 0.05 vs. Ang II group; ^#^ *p* < 0.05 vs. Ang II/shNEAT1 group. Data represent mean ± SD from three independent experiments.

To determine whether NPAS2 contributes to the effect of NEAT1 on cardiac fibroblasts, Ang II treated cardiac fibroblasts were co-transfected with shNEAT1 and NPAS2. The cardiac fibroblasts viability, migratory ability, and collagen production ability in co-transfected shNEAT1 and NPAS2 cells were greater than those in cardiac fibroblasts transfected with shNEAT1 (Figures 4A–F). These findings indicated that NPAS2 is a functional target of NEAT1 and that NPAS2 eliminates the suppressive effect of NEAT1 inhibitors on cardiac fibroblasts.

### NEAT1 Knockdown Attenuates Ang II Caused Atrial Fibrosis *In Vivo*

**FIGURE 5:**
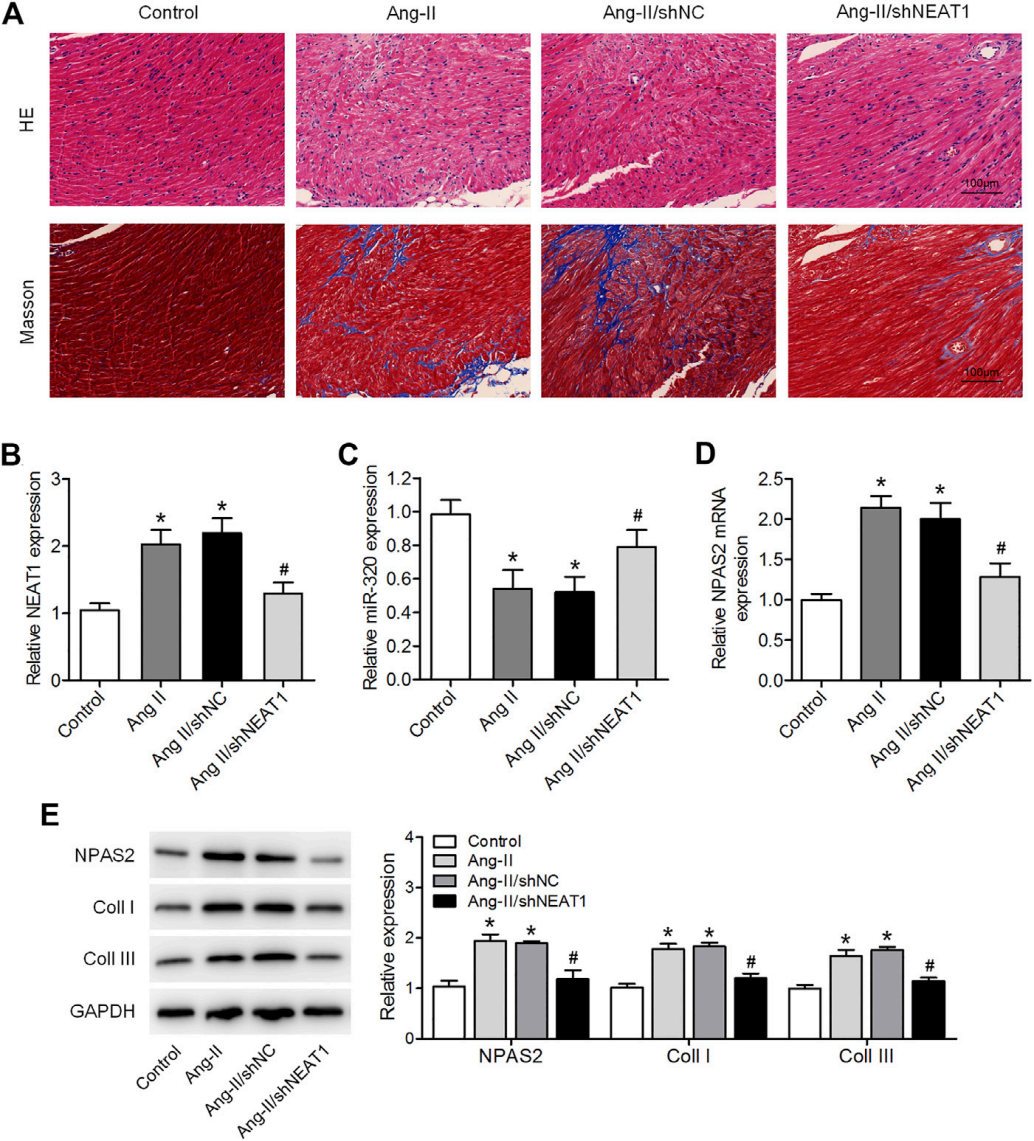
NEAT1 knockdown attenuates the Ang II-induced atrial fibrosis *in vivo*. **(A)** HE and Masson’s trichrome stainings of murine right atrial tissues. **(B,C)** NEAT1 and miR-320 expressions in right atrial tissues from each group were detected using qRT–PCR. **(D)** NPAS2 mRNA expression in right atrial tissues from each group was determined. **(E)** NPAS2, coll I, and coll III protein expressions in right atrial tissues from each group were detected. *p < 0.05 vs. control group; ^#^ *p* < 0.05 vs. Ang II/shNC group. Data represent mean ± SD from three independent experiments.

We further affirmed the function of NEAT1 in atrial fibrosis through *in vivo* experiments. HE and Masson’s trichrome stainings indicated a disarray of myocardial fibers, expanded nuclear spacing, and increased atrial fibrosis in the Ang II induced group, while NEAT1 deletion suppressed the Ang II caused inflammatory cell infiltration and atrial fibrosis (Figure 5A). We found that NEAT1 and NPAS2 expressions were increased, whereas miR-320 expression was decreased in right atrial tissues from the Ang II group than those in the control group, and these expressions were reduced in the Ang II/shNEAT1 group (Figures 5B–E). Moreover, Ang II injection enhanced the protein expressions of coll I and coll III in right atrial tissues, whereas deletion of NEAT1 attenuated the Ang II caused collagen production (Figure 5E).

## Discussion

Despite a mass of evidence demonstrated the function of atrial fibrosis in AF (Xu et al., 2018), data on fibrotic processes in AF are limited. Here, we showed that NEAT1 was increased and positively related to coll I and coll III levels in right atrial tissues of patients with AF. NEAT1 knockdown reduced Ang II caused cardiac fibroblast proliferation, migration, and collagen production *in vitro* and attenuated Ang II caused murine atrial fibrosis *in vivo*. Moreover, we demonstrated that NEAT1 exerted its effects by functioning as a miR-320 ceRNA to modulate NPAS2 level.

Increasing evidence has revealed that atrial fibrosis contributes to the pathological process of AF and that atrial fibrosis suppression could be a reasonable approach for AF prevention and treatment (Chang et al., 2017). Recent research has shown that lncRNA NEAT1 is participated in the pathogenesis of different diseases, including fibrosis. NEAT1 is upregulated in CCl4 caused liver fibrosis, and it accelerates liver fibrosis progression (Yu et al., 2017; Kong et al., 2019); NEAT1 knockdown in HK2 cells inhibited the renal fibrosis-related markers TGF-β1 and CTGF (Yang et al., 2020). Furthermore, NEAT1 is indispensable for fibroblast and cardiomyocyte survival and affects fibroblast functions (Kenneweg et al., 2019). We speculated that NEAT1 contributes to atrial fibrosis. Here we observed that NEAT1 expression was increased and was positively related to coll I and coll III levels in the right atrial tissues of patients with AF. Cardiac fibroblast proliferation, migration, and differentiation play key roles in the pathogenesis of atrial fibrosis and structural remodeling in AF patients (Porter and Turner, 2009; Xu et al., 2018). Here a cell model of atrial fibrosis was achieved in Ang II-induced atrial fibroblasts. We found that Ang II enhanced NEAT1, coll I and coll III levels and promoted cardiac fibroblast proliferation and migration, whereas these effects were reduced by NEAT1 knockdown. We further confirmed this result in an Ang II induced atrial fibrosis mouse model.

Several existing studies have indicated that lncRNAs participate in the ceRNAs regulatory network to negatively regulate the miRNAs expression (Yan et al., 2018), such as NEAT1. For instance, NEAT1 functions as a sponge for miR-365a-3p to facilitate gastric cancer progression through targeting ABCC4 (Gao et al., 2020); furthermore, NEAT1 sponges miR-129 to regulate renal fibrosis via modulating coll I (Li et al., 2020). To determine the mechanism of NEAT1 regulation in AF, we performed bioinformatics databases and discovered that NEAT1 shared miR-320 response element with NPAS2. Several studies have shown that miR-320 is associated with heart-related diseases. For example, miR-320 participates in the cardioprotective effect of insulin against myocardial ischemia via downregulating survivin (Yang et al., 2018). The protective effect of miR-320 has also been shown in ventricular remodeling after myocardial ischemia–reperfusion injury (Song et al., 2014). Importantly, exosomal miR-320 derived from adipose tissue-derived mesenchymal stem cells suppresses apoptosis of cardiomyocytes in AF patients (Liu et al., 2019). Our study demonstrated that Ang II decreased miR-320 expression, whereas NEAT1 knockdown increased it. NPAS2-deficient fibroblasts expedite skin wound healing and dermal collagen reconstruction (Sasaki et al., 2020), and NPAS2 promotes liver fibrosis via direct transcriptional activation of Hes1 in hepatic stellate cells (Yang et al., 2019). In our study, we demonstrated that miR-320 overexpression suppressed NPAS2 expression and miR-320 inhibition reversed the suppressive effect of shNEAT1 on NPAS2 expression. Moreover, we determined that NPAS2 overexpression reversed the effects of NEAT1 knockdown on Ang II caused murine cardiac fibroblast proliferation, migration, and collagen production.

In conclusion, our research provides evidence that NEAT1 knockdown inhibits cardiac fibroblast proliferation, migration, and collagen production as well as mice atrial fibrosis via regulating the miR-320–NPAS2 axis, suggesting that NEAT1 is a novel molecular target for AF treatment.

## Data Availability

The raw data supporting the conclusion of this article will be made available by the authors, without undue reservation.
